# Fabrication of complex optical phantoms using on-the-fly multi-filament mixing 3-D printing

**DOI:** 10.21203/rs.3.rs-5500473/v1

**Published:** 2024-12-11

**Authors:** Rahul Ragunathan, Miguel Mireles, Edward Xu, Aiden Lewis, Morris Vanegas, Qianqian Fang

**Affiliations:** 1 Department of Bioengineering, Northeastern University, Boston, 02115, USA

## Abstract

We report a method to directly 3-D print complex heterogeneous optical phantoms with programmable tissue-mimicking absorption and scattering properties. The proposed approach utilizes commercially available multi-color mixing extruders and off-the-shelf polylactic acid (PLA) filaments, making this technique low-cost and broadly accessible. We systematically characterized optical properties, including both absorption and reduced scattering coefficients, at a wide range of mixing ratios of gray, white and translucent filaments and validated our hypothesis of a linear-mixing model between the filament mixing ratios and the resulting optical properties. Various techniques were used to design and fabricate sophisticated solid phantoms, including the design of color-purging towers, and the optimization of several printing parameters to improve print quality. To demonstrate the feasibility of this technique for generating anatomically complex phantoms with tunable optical absorption and scattering properties within tissue-relevant ranges, we designed and fabricated three heterogeneous optical phantoms. One of the presented phantoms was specifically designed to support quality assurance efforts in evaluating diffuse optics instruments and methodologies across various institutions. We have characterized the printed phantoms and observed an average error between 12%−15% compared to our linear-mixing model predicted values.

## 1 Introduction

Diffuse optical imaging (DOI) techniques have been active areas of research in recent decades due to their capabilities to quantify physiological contrasts from biological tissues centimeters below the skin using non-ionizing near-infrared (NIR) light^1^. These approaches have attracted significant interest due to potential clinical applications ranging from functional brain imaging^2–4^ to breast cancer diagnosis^5–7^. The success of many of the DOI techniques depends on accurate quantification of wavelength-dependent optical absorption and scattering properties, which permit the estimation of chromophore concentrations, such as oxy-hemoglobin, deoxy-hemoglobin, water and lipid content, to characterize the physiology of interrogated tissue samples^8,9^. To assist in developing and characterizing DOI imaging systems, many diffuse optics research groups have investigated robust methods for fabricating optical phantoms, i.e. a durable material with predictable tissue-mimicking optical properties or complex anatomical shapes^10–14^. The utilization of optical phantoms is thus both crucial and necessary in ensuring the validity of measured results from DOI systems. However, most of these previously reported efforts were initiated by individual research laboratories for the purpose of internally characterizing and validating instrument performance as well as for calibrating real-world DOI measurements^10,15^.

Optical phantoms are typically made by mixing a base material with absorbing pigments, such as India ink or carbon black powder, and scattering pigments, such as intralipid or metal oxides^13,15–20^. Depending on the base material, optical phantoms are typically classified as liquid^13,15,17–20^ or solid^21–24^ phantoms. Liquid phantoms are usually easier and faster to fabricate but offer limited durability, often requiring refrigeration to extend their shelf-life beyond single experimental use^25^. Solid phantoms, on the other hand, are typically made of durable materials such as silicone rubber, epoxy resin or other resins that have a stable chemistry and therefore do not require special maintenance^26–28^. This allows them to be used repeatedly for extended period of time. As a result, solid phantoms have received widespread adoption among research labs. Although phantom fabrication procedures have been reported in previous publications^29,30^, many of these procedures require precise measurements of pigment quantities and lengthy curing processes, which are often prone to operator variability, making them challenging to reproduce outside of the originating lab. In recent years, a growing community movement towards standardizing DOI measurements across multiple institutes has led to the collaboration of various consortia^31^. However, most of these initiatives designated a centralized facility or company to fabricate and distribute phantoms across the consortium^32^. While this centralized approach ensures more consistent quality control of materials and fabrication procedures, it does not directly address challenges related to high manufacturing and distribution costs or the issue of phantom reproducibility between labs. As such, a simple, reproducible and, more importantly, widely accessible optical phantom fabrication approach is still critically needed for the DOI research community.

In addition, the majority of published DOI optical phantoms, including many commercially available standardized phantom sets, utilize homogeneous phantoms made of a single material with simplistic geometries^29,33^. While these phantoms are relatively easy to produce and suitable for basic spectroscopic measurement characterization, they lack the anatomical complexity necessary for systematically evaluating DOI systems that are designed for resolving heterogeneous structures, such as most diffuse optical tomography (DOT)^1,3^ and spatial frequency-domain imaging (SFDI)^34^ devices. Specifically, the use of phantoms with simple geometries such as slabs or semi-spheres may result in calibration errors due to the mismatch between phantom shape and actual anatomical shapes. The fabrication and use of heterogeneous optical phantoms are only reported in limited studies^14,35,36^. Some of these reported complex heterogeneous phantom fabrication procedures require complex processes, including multiple casting and molding steps ^14,37^. Developing reliable methodologies that are capable of fabricating both homogeneous and complex heterogeneous phantoms is expected to greatly facilitate the development of next-generation DOI systems.

Three-dimensional (3-D) printing has recently been utilized as a valuable tool for the manufacturing of sophisticated medical phantoms^24,38,39^. Early works on using 3-D printing techniques in developing optical phantoms have included the use of fused deposition modeling (FDM)^40–42^, stereolithography (SLA)^36,39,43–45^ and spin-coating^46^ for fabricating phantoms with complex structures. While these methods show promising results, they also face major limitations. For instance, Dong *et al.*^41^ reported a custom-built wax-resin based FDM 3-D printer that can programmatically mix scattering and absorbing agents using a custom extruder. While this approach is able to print heterogeneous phantoms, it relies on a specialized printer that is difficult replicate among other labs; the produced phantoms also exhibit limited inclusion resolution due to the specific wax material used. In 2015, Diep *et al.* ^40^ also reported a method for fabricating heterogeneous phantoms by using custom-manufactured acrylonitrile butadiene styrene (ABS) filaments from resins with tuned optical properties and a widely available, off-the-shelf dual extruder FDM printer. However, the need to manually fabricate specialized filaments from resins, along with the limitation of accommodating only up to two optical properties, hampers its potential for widespread adoption. More recently, Ruiz *et al.*^43^ reported an SLA based approach by adding fluorophores, absorbing agents and scattering agents to the curing resin, allowing for the generation of luminescence optical phantoms with complex shapes. Nevertheless, this approach can only create prints made of a single homogeneous material.

Developing a more versatile and accessible method for fabricating heterogeneous optical phantoms could significantly advance standardization efforts in DOI instrumentation. This improvement would expand applications beyond spectroscopic uses to more advanced imaging modalities, including various DOT and SFDI systems, where the characterization of complex domains and quantitative imaging quality metrics, such as spatial resolution and contrast, is essential. Standardized quality assurance (QA) phantoms, such as those widely adopted in established imaging modalities, including x-ray and mammography^47^ still do not currently exist among many DOT or SFDI applications. While commercial services are available and capable of generating customized DOT phantoms with user-defined inclusions, the high cost, low accessibility and limited phantom complexity present major barriers for broad adoption among the DOI communities. Thus, a reliable DOT phantom fabrication method that is widely accessible and reproducible across labs would enable the DOT research community to start the conversation in designing QA phantoms for characterizing and comparing diverse DOT imaging systems.

In this work, we report on a versatile optical phantom fabrication technique utilizing multi-filament 3-D printing to programmatically mix absorbing (gray), scattering (white) and translucent off-the-shelf polylactic acid (PLA) filaments on-the-fly to produce complex heterogeneous phantoms^48,49^. Our approach can directly utilize commercially available and low-cost multi-filament fusion extruders and commonly used FDM filaments, thus having the potential to be widely accessible and easy to implement. Compared to previous 3-D printing based optical phantom fabrication methods, our technique does not require laborious methods prior to printing and is capable of generating arbitrarily complex 3-D solid phantoms with an unlimited number of unique optical properties within a contiguous phantom.

In the following sections, we begin by detailing the key parameters of our 3-D printing approach, including the printer specifications, the filaments used, and our optimized printing, purging, and post-processing protocols. In the following Results section, we report the results for a multi-filament titration study, by printing uniform optical phantom blocks at a wide range of mixing ratios and subsequently measuring the resulting phantom’s optical properties. Using the measured optical properties,we further validate our assumed linear-mixing-model between the resulting optical properties and filament mixing ratios. Additional homogeneous phantoms are printed at novel mixing ratios and are measured to compare with the predicted optical properties from the characterized linear-mixing model for the chosen filaments. Next, we showcase our ability to generate sophisticated heterogeneous phantoms comprising complex structures by generating a segmented mouse phantom derived from the Digimouse atlas^50,51^, a prototype DOI QA phantom with 5 unique mixing ratios, as well as a more sophisticated DOT phantom with 16 inclusions made of 8 unique mixing ratios. Finally, we outline the main strengths and limitations of the current approach in the Discussion section.

## 2 Methods

### 2.1 Multi-filament extruder and 3-D printer

**Figure 1.**
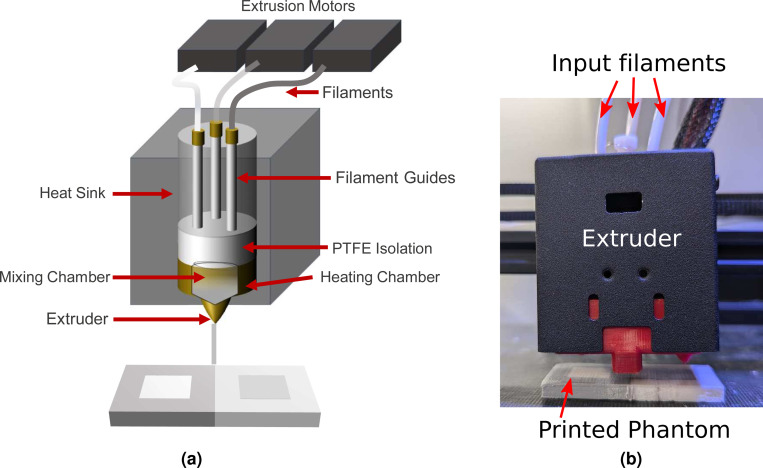
Filament-mixing extruder schematic diagram and photograph. In the schematic shown in (a), we show three programmable extrusion motors corresponding to the gray, white and translucent filaments, along with the filament guides, the heating and mixing chambers. In (b), we show a color photo of the ZoneStar M4V5 extruder fed with 3 input filaments. A photo of a printed heterogeneous phantom is also shown in (b).

All printed models were created by mixing commercially available gray (model # PLA175RGY, 3-D Solutech, Seattle, USA), white (model # ST175CLPLA) and natural clear (model # PLA175RWHT) 1.75 mm PLA filaments using a ZoneStar M4V5 4-filament mixing extruder head on a ZoneStar Z9V5 Pro 3-D printer (ZoneStar, Shenzhen, China). The extruder utilizes a 0.6 mm nozzle size to achieve improved reliability. In addition, all samples were printed with a layer-thickness of 0.2 mm to yield a smooth surface appearance and satisfactory spatial resolution without incurring lengthy printing times. A line width (spacing between two adjacent horizontal extruding paths) of 0.3 mm and an 90-degree alternating unidirectional infill pattern is used. A maximum printing speed of 22 mm/s was chosen to minimize chances of mechanical failures while ensuring minimal visual artifacts. The nozzle schematic, a photo of the M4V5 printer nozzle, as well as the extrusion motors and filament can be seen in Fig. 1. We currently use 3 out of the 4 filament inlets supported by the extruder. The unused filament channel can be used to accommodate other filament types, such as water-soluble filaments as supporting materials for printing complex shapes, or fluorescent or phosphorescent light-emitting filaments for creating luminescent optical phantoms as reported in our prior work^52^.

We want to note that although this reported work has been tested using the aforementioned extruder and filament models, our printing and characterization methods described below are expected to be readily generalizable towards other multi-filament printers and filaments. When a different printer and filament brands or materials are used, one could potentially repeat the titration study outlined in Section 2.5 below and use the measured optical properties to create 3-D optical phantoms similar to our reported samples.

### 2.2 Special considerations for enabling on-the-fly filament-mixing

A number of unique characteristics must be carefully considered when fabricating optical phantoms using 3-D printing-based approaches. First, unless empty air cavities are desired, optical phantoms are usually printed with solid and uniform infill patterns with minimal air gaps between extruding paths. To avoid trapping air pockets in a solid phantom print, one should choose a high infill ratio in the printer setting, as oppose to a sparse infill ratio used in typical 3-D printing tasks. The infill ratio can be adjusted using the slicer tool – a 3-D printing software typically used to convert a 3-D surface mesh model into step-by-step motor movement instructions, referred to as the G-code, along each thin layer of the z-axis of the printing volume. If supported by a slicer software, an infill ratio greater than 100% is recommended to inform the filament extruders to feed excessive filaments to the extruder to effectively fill the microscopic air gaps between adjacent extruded mixed filament paths. Slightly raising the extruder temperature above the filament’s recommended temperature range is also found to be helpful in minimizing potential air gaps within the printed object. In this work, we use an open-source slicer, Cura (v4.12.1), with an experimentally optimized infill ratio of 110% and an extruder temperature at 205°C to achieve quality printing results.

Extruder purging is a crucial step in 3-D printing of multi-material objects. The purging step removes the melted filaments of previously used mixing ratio when switching from one mixing ratio to another within the same z-slice. When N number of materials, characterized by N sets of unique mixing ratios, present along any z-slice of the print, the extruder must print each single-material region sequentially, and purge its contents before printing the next material at a different mixing ratio. In this work, purging is achieved by running a modified G-code postprocessing Python scripts (initial version provided by ZoneStar) or by manually creating secondary models outside of the primary printed model. When the purging instructions are being executed, the extruder is moved to an area outside of the primary model space and printed as an additional structure, referred to as the purging tower, using the residual materials inside the extruder while switching to the new mixing settings. The size of the purging tower can be adjusted to ensure the thoroughness of the color switching.

Multiple purging tower designs have been explored. In this work, a range of purging towers sizes of x/y dimensions 15×15 mm^2^ – 15×35 mm^2^ were used for all prints by placing a series of purge towers corresponding to each material on the side of the primary print. The number of purging powers is set to the number of unique mixtures within a single contiguous model. Typically, a larger purging tower size is required for more accurate transitions between different filament mixing combinations but can increase the total print time, which could adversely increase the risk of print mechanical failure. If a print contains only a uniform mixing ratio beyond a specific z-slice, no additional purging is required beyond the last heterogeneous z-slice. Furthermore, within the slicer, the purging towers must be carefully placed alongside the primary model such that the extruder purges each material prior to transitioning to continue printing the primary model for every layer of the print. While purging adds extra printing time and material consumption, it is an essential step in achieving both accurate control of optical properties while also generating clear boundaries between heterogeneous regions within the print.

Frequent transitions between the purging tower and model within each z-slice can adversely impact the quality of the print due to the “stringing” effect of extruder movements. One should minimize the needs and frequencies of purging by adjusting the orientation of the print while considering the relative positions of the inclusions.

A number of additional settings have also been optimized to improve phantom printing quality. For example, a “z-hop” height of 0.3 mm was used when switching between different mixing combinations to prevent the extruder from colliding with printed parts when the nozzle moves between the purge tower and the printed model. In addition, a Python post-processing script was applied to restrict nozzle movements along the x and y orthogonal directions only, thus minimizing diagonal movements, further minimizing printing artifacts. Finally, we apply light sanding of the print surfaces using a belt-disk sander, followed by simply washing and drying the model to remove the uneven surfaces caused by the extruder movements between the print and the purging tower.

### 2.3 Optical properties characterization

For conveniently measuring localized optical properties of our printed optical phantoms, we have built and tested a standard 3-phase spatial frequency domain imaging (SFDI) system^34,53^. This SFDI system consists of a digital-mirror-device (DMD) based projector (AAXA Tech, USA) illuminated by a red light-emitting-diode (LED) at 635 nm, and a complementary metal-oxide-semiconductor (CMOS) camera (FLIR, USA) as the detector. A pair of cross-polarizers are placed in front of the camera and projector to minimize the effect of specular refection. Spatial frequencies of 0 and 0.1 mm^−1^ are used for all measurements. The sample optical properties, specifically, absorption coefficients $\mu_{a}$ (mm^−1^) and reduced scattering coefficient $\mu_{s}^{\prime }$ (mm^−1^), at each camera pixel are estimated using look-up tables generated (LUT) based on White Monte-Carlo (WMC) techniques^54,55^ using our in-house Monte-Carlo simulator, Monte Carlo eXtreme (MCX)^56^, with a refractive index (n) of 1.4 and anisotropy (g) of 0.9^57^. The SFDI data acquisition and processing were controlled using custom software written in MATLAB (MathWorks, Natick, USA). The projector illumination area is approximately 14×9 cm^2^ in size with a camera per-pixel area of roughly 0.035 mm^2^. A homogeneous slab-shaped silicone phantom is used as the calibration phantom for all SFDI measurements. The calibration phantom has measured optical properties (λ=635 nm) of $\mu_{a}=0.0064$ mm^−1^ and a $\mu_{s}^{\prime }=1.42$ mm^−1^ obtained by a commercial frequency-domain near-infrared spectrometer (ISS, Champaign, IL).

### 2.4 Linear-mixing model

The combined absorption coefficient in a mixture of chromophores is represented by the Beer’s law^1^, where the total absorption is a linear combination of each individual chromophore’s extinction coefficient multiplied by its concentration or volume fraction^15^. Here we hypothesize that the optical properties of the mixed-filament prints are related to the fractions of each filament type extruded in a given filament mixture. The assumed linear-mixing relationship can thus be defined as

(1) $$\mu =A\times R_{g}+B\times R_{w}+C,$$

where μ represents either $\mu_{a}$ or $\mu_{s}^{\prime }$; $R_{g}$ and $R_{w}$ represent the percentage filament mixing ratios (with values between 0 and 100) of gray and white filaments, respectively; $100-\left(R_{g}+R_{w}\right)$ represents the volume percentage fraction (i.e. $R_{t}$) of the translucent filament; A, B, C are scalar linear coefficients. For simplicity, hereinafter, we use the notation $\left(R_{g}/\text{G},\;R_{W}/\text{W},\;R_{t}/\text{T}\right)$ to represent the filament mixing configurations in all prints below.

### 2.5 Validation of linear-mixing model with a titration study

In order to systematically characterize the effect of filament mixing on optical properties while also validating our above hypothesized linear-mixing model, we have 3-D printed a set of 16 cubic prints, each with a size of 1.5×1.5×2 cm^3^ and configured at a unique mixing ratio of gray/white/translucent filaments. The mixing ratios of the 16 prints are reported in Table 1a. To minimize 3-D printer systematic offsets due to switching between printing tasks and mixing ratios, we group every four cubic prints into a single 1.5×2×6 cm^3^ “ruler” and print each ruler vertically (i.e. along the z-axis). This configuration minimizes the effect of purging volume throughout the printing process, and allows for a more uniform color constant throughout the full length (1.5 cm) of each segment. Printing each of the rulers takes roughly 2.5 hours to complete on the ZoneStar printer.

**Figure 2.**
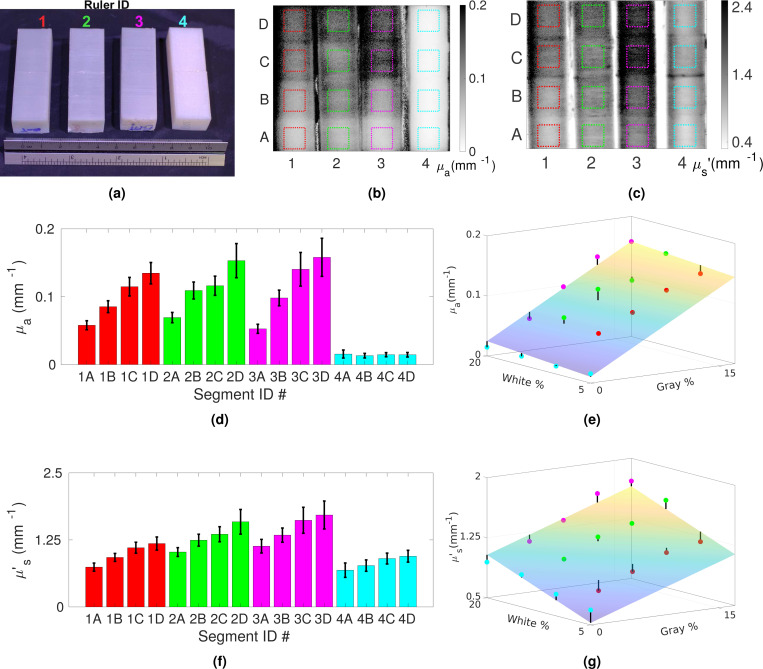
Titration study characterizing relationships between output optical properties and filament-mixing ratios. A color image of the 4 titration rulers (1–4, color-coded) is shown in (a). Recovered $\mu_{a}$ and $\mu_{s}^{\prime }$ images from SFDI measurements of the 4 rulers can be seen in (b) and (c), respectively. Each ruler contains 4× segments printed with different mixing ratios, each uniquely labeled by a segment ID (A-D); the median and standard deviation for $\mu_{a}$ and $\mu_{s}^{\prime }$ computed across each segment’s ROI, shown in dashed squares, are summarized in (d) and (f) respectively following the ruler-segment ID notation. The fitted planes for $\mu_{a}$ and $\mu_{s}^{\prime }$ are shown in (e) and (g), respectively, with errors to the fitted planes marked by a short vertical line.

To characterize the optical properties of the printed samples, we use our SFDI device to measure all 4× rulers (denoted as 1–4 in Table 1a) in a single acquisition. The SFDI topographic maps of the recovered $\mu_{a}$ and $\mu_{s}^{\prime }$ images can be found in Figs. 2b and 2c. For each print, we select a region-of-interest (ROI) of roughly 0.7 cm^2^ from the SFDI $\mu_{a}$ and $\mu_{s}^{\prime }$ images, from which the median $\mu_{a}$ and $\mu_{s}^{\prime }$ values are obtained and used to perform a linear regression according to the hypothesized linear-mixing model shown in Eq. 1.

To further test the robustness of the fitted linear-mixing models, we printed five additional 1.5×2×1.5 cm^3^ models, shown as V-1 to V-5 in Table 1b. Each block was printed at a novel mixing ratio setting without overlapping with the previously measured 16× titration samples. We then use the above estimated linear-mixing model to predict the anticipated $\mu_{a}$ and $\mu_{s}^{\prime }$ based on the titration data points and compare those with the SFDI measured values. In order to assess the accuracy of the fitted linear-mixing model, a relative error ε is computed.

(2) $$\varepsilon =\frac{\left|\mu_{measured}-\mu_{predicted}\right|}{\mu_{predicted}}$$

where $\mu_{measured}$ corresponds to the median $\mu_{a}$ or $\mu_{s}^{\prime }$ value estimated from all SFDI measurement pixels in a given region of interest (ROI) while $\mu_{predicted}$ corresponds to the predicted $\mu_{a}$ or $\mu_{s}^{\prime }$ value based on the fitted linear-mixing model.

### 2.6 Filament mixing performance characterization

To further assess the capability of creating spatially tunable optical properties, we designed three simple benchmark models, as illustrated in Fig. 4. Specifically, the first benchmark model (Benchmark #1, see Fig. 4a) consists of three 1.5×1.5×0.5 cm^3^ solid segments, with the respective filament mixing ratios (100/G, 0/W, 0/T), (50/G, 50/W, 0/T) and (0/G, 100/W, 0/T) shown from left to right, respectively. This test is used to demonstrate the printer’s capability of changing mixing ratios along a single axis. The second benchmark (Benchmark #2, see Fig. 4b) consists of two adjacent 3×3×0.5 cm^3^ square frames, each embedded with a concentric 1.5 × 1.5 × 0.5 cm^3^ square-shaped inclusion. The square-frame and the inclusion have mixing ratios (100/G, 0/W, 0/T) and (0/G, 100/W, 0/T) and are arranged in the inverted order between the two adjacent square-frames. This benchmark is used to demonstrate the capability of creating embedded structures. The third benchmark (Benchmark #3, Fig. 4c) consists of a 3×3 tiles of 1.5×1.5×0.5 cm^3^ square solid segments each with a unique filament mixing combination. This benchmark is used to demonstrate the printer’s ability to create two-dimensional heterogeneous structures with distinct mixing ratios.

### 2.7 Fabricating complex-shaped heterogeneous phantoms

To demonstrate the ability to fabricate optical phantoms with complex 3-D biological tissue shapes and spatially varying optical contrasts, we design a sample print derived from the head and torso section of the Digimouse atlas^50^. A schematic of this phantom can be found in Fig. 5a. For simplicity, we create this phantom with three distinctive tissue types to mimic the mouse brain, lungs and the surrounding soft tissues. Although our printer is capable of printing the tissue regions to match their literature $\mu_{a}$ and $\mu_{s}^{\prime }$ values, for the purpose of facilitating visual assessment of the prints, we artificially exaggerated the literature $\mu_{a}$ contrasts by 5-fold and that of $\mu_{s}^{\prime }$ by 2-fold in both the brain and lung regions. The surrounding soft-tissue is printed using literature reported values at 750 nm for muscle-tissue optical properties with a targeted $\mu_{a}=0.01$ mm^−1^ and $\mu_{s}^{\prime }=0.5$ mm^−1 58^, resulting in a mixing ratio of (0/G, 5/W, 95/T). For the brain region, we use a mixing ratio of (26/G, 40/W, 34/T) to target at the 5-fold exaggerated $\mu_{a}=0.245$ mm^−1^ and 3-fold exaggerated $\mu_{s}^{\prime }=3.52$ mm^−1^, estimated based on the literature gray-matter (GM) and white-matter (WM) optical properties^59^ with a 80:20 GM-to-WM mass ratios^60^. For the lung region, we use a mixing ratio setting of (4/G, 11/W, 49/T) to target at the respectively scaled $\mu_{a}=0.38$ mm^−1^ and $\mu_{s}^{\prime }=2.18$ mm^−1^ based on literature optical properties^61,62^.

### 2.8 Prototype quality assurance phantom for transmission and reflection DOI systems

Inspired by the design of the standardized American College of Radiology (ACR) mammography quality assurance (QA) phantom^47^, we have designed a 9 × 9 ×0.5 cm^3^ slab phantom containing 4× bar- and 4× cylindrical-shaped inclusions with various sizes and contrasts. All inclusions have a thickness of 1 mm and are located at the bottom face of this phantom. The remaining space is printed with a uniform background material, with a target optical property $\mu_{a0}=0.019$ mm^−1^ and $\mu_{s0}^{\prime }=0.83$ mm^−1^, designed to mimic the skull and scalp tissue at 630 nm^63^. A diagram showing the dimensions and the target optical properties of this prototype QA phantom is shown in Fig. 6a. The 3 disk inclusions (C1-C3) along the top row share the same optical properties ($15\times \mu_{a0}$ and $3\times \mu_{s0}^{\prime }$), with diameters 1.5 cm, 1.0 cm and 0.5 cm, respectively; a more absorbing 0.5 cm diameter disk-inclusion (C4) is positioned at the bottom-right corner ($25\times \mu_{a0}$ and $3\times \mu_{s0}^{\prime }$). Two pairs of 0.5 × 2 cm^2^ bar-shaped inclusions are printed along the bottom section, sharing a $2\times \mu_{s0}^{\prime }$ scattering coefficients. One of the pairs is about half of the $\left(6\times \mu_{a0}\right)$ absorption compared to the other pair $\left(12\times \mu_{a0}\right)$. This phantom can be used for characterizing imaging spatial resolution and contrast recovery for diverse DOI systems in both reflection and transmission settings when measured from either side of the phantom.

### 2.9 Complex heterogeneous phantoms for tomographic imaging settings

To further push the boundary of this technology, we have designed an even more sophisticated 10× 1.5 × 5 cm^3^ heterogeneous DOT phantom that contains 8× unique sets of target optical properties and 16× inclusions. The printing volume of this phantom is also nearly doubled compared to the QA phantom described above, allowing us to “stress-test” the printer and understand its printing stability. The detailed schematic of the phantom as well as the optical properties of all embedded inclusions are shown in Fig. 7a. All inclusions, except the two largest cylindrical inclusions, extend throughout the full depth (15 mm) of the phantom; the end-face of the largest cylindrical inclusion is 2 mm below the phantom surface, while the second largest cylindrical inclusion is 1 mm below the phantom surface.

## 3 Results

### 3.1 Titration phantom characterization

A photo of the 16× titration prints, as detailed in Section 2.5, is shown in Fig. 2a. The recovered $\mu_{a}$ and $\mu_{s}^{\prime }$ maps, derived from SFDI measurements, are shown in Figs. 2b and 2c, respectively. To minimize the boundary effect of SFDI, for each sample, we crop a squared shaped ROI of 45-by-45 pixels (0.7 cm^2^) from the central portion of each homogeneous segment, shown as dashed-line squares in both of these images. The median values of the estimated $\mu_{a}$ and $\mu_{s}^{\prime }$ within these ROIs are plotted in Figs. 2d and 2f, respectively, with error bars denoting the standard deviations between pixel-wise optical properties within each ROI. The overall range of the estimated optical property values extends between 0.013 mm^−1^ and 0.15 mm ^−1^ for $\mu_{a}$ and between 0.69 mm^−1^ and 1.72 mm^−1^ for $\mu_{s}^{\prime }$, covering those typically found in biological tissues^9^.

In Figs. 2e and 2g, we plot the measured $\mu_{a}$ and $\mu_{s}^{\prime }$ values, respectively, against the filament mixing ratios, $R_{g}$ as the x-axis and $R_{w}$ as the y-axis used in each print. A linear plane fitted using Eq. 1 is also obtained and shown in these plots. For $\mu_{a}$, the fitted linear-mixing model is found to be $\mu_{a}=0.0078\times R_{g}+0.00095\times R_{w}+0.0056$ (mm^−1^) with an $R^{2}=0.97$. Similarly, the fitted linear-mixing model for $\mu_{s}^{\prime }$ is $\mu_{s}^{\prime }=0.036\times R_{g}+0.034\times R_{w}+0.35$ (mm^−1^), with an $R^{2}=0.92$. The fitted linear coefficients suggest that $\mu_{a}$ is primarily tuned by modulating the fraction of gray filament $\left(R_{g}\right)$; however, the white and gray filaments appear to contribute roughly equally to the model estimated $\mu_{s}^{\prime }$ value while the translucent filament sets a theoretical lower-bound of $\mu_{s}^{\prime }$ value roughly at 0.35 mm^−1^.

### 3.2 Further validation of the linear-mixing model

**Figure 3.**
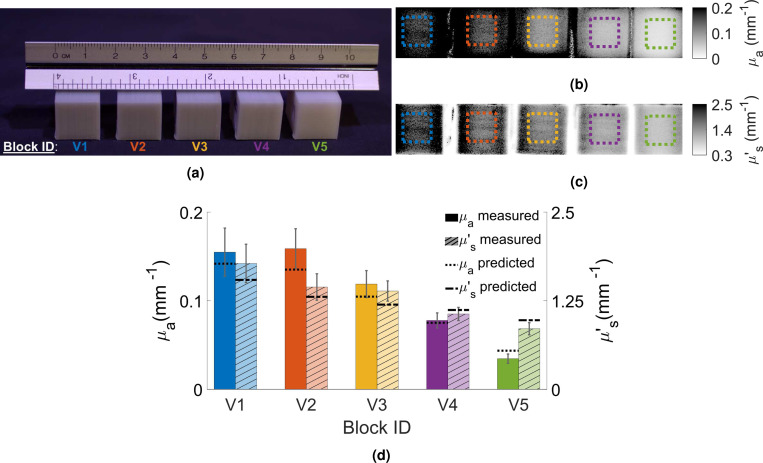
Measured optical properties for the 5× (V-1 to V-5) validation phantoms. A color photo of these printed phantoms is shown in (a). The SFDI-measured $\mu_{a}$ and $\mu_{s}^{\prime }$ maps are shown in (b) and (c), respectively, with color-coded ROIs shown as dashed-box overlays. The measured median $\mu_{a}$ and $\mu_{s}^{\prime }$ from are shown in (d) as solid and hatched bars, respectively, with error bars denoting the standard deviations. The dotted and dashed horizontal lines are predicted estimates using the fitted linear-mixing model from the titration study.

A photo of the 5× validation prints (V-1 to V-5) is shown in Fig. 3a. The SFDI-measured $\mu_{a}$ and $\mu_{s}^{\prime }$ topographic images are shown in Figs. 3b and 3c, respectively. Similar to the previous section, we also manually select square-shaped ROIs of roughly 0.7 cm^2^ for each print, shown in dashed line overlays in the SFDI images. The estimated medians for $\mu_{a}$ (hatch-filled) and $\mu_{s}^{\prime }$ (solid) from these ROIs are plotted as grouped bar plots, with standard deviations shown as error bars, in Fig. 3d. The predicted $\mu_{a}$ and $\mu_{s}^{\prime }$ values based on the fitted titration data (see Fig. 2), are also shown as horizontal lines. The average percentage errors for $\mu_{a}$ is approximately 12.89% and 11.82% for $\mu_{s}^{\prime }$ among these 5 validation prints compared to their corresponding linear-mixing model estimates.

### 3.3 Printer filament-mixing tests

**Figure 4.**
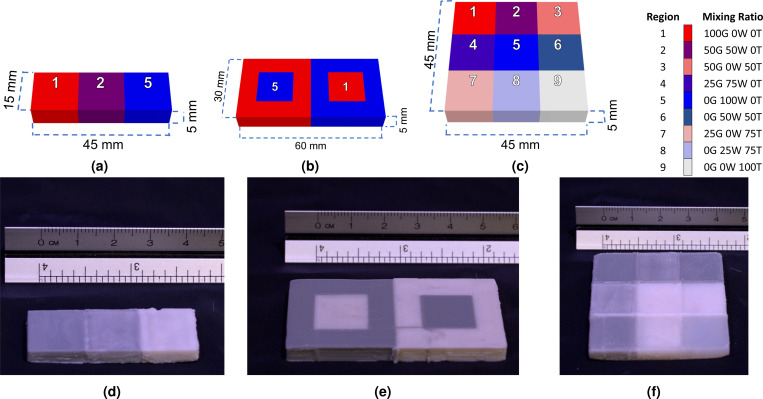
Dimensions and photos of the fabricated filament-mixing test phantoms. The schematics of the three printed benchmarks are shown in (a)-(c) with each unique material labeled from 1 to 9. The corresponding filament mixing ratios are shown in the legend. The corresponding photo of each benchmark is shown in (d)-(f).

The schematics and photos of the 3× simple filament-mixing test prints (Benchmarks 1–3) described in Section 2.6 are shown in Fig. 4. The printing time for Benchmarks 1, 2 and 3 are approximately 1.5 hours, 2.25 hours and 4.5 hours, respectively; the total filament consumption is approximately 12 grams, 18 grams, and 36 grams, respectively.

### 3.4 Digimouse phantom

**Figure 5.**
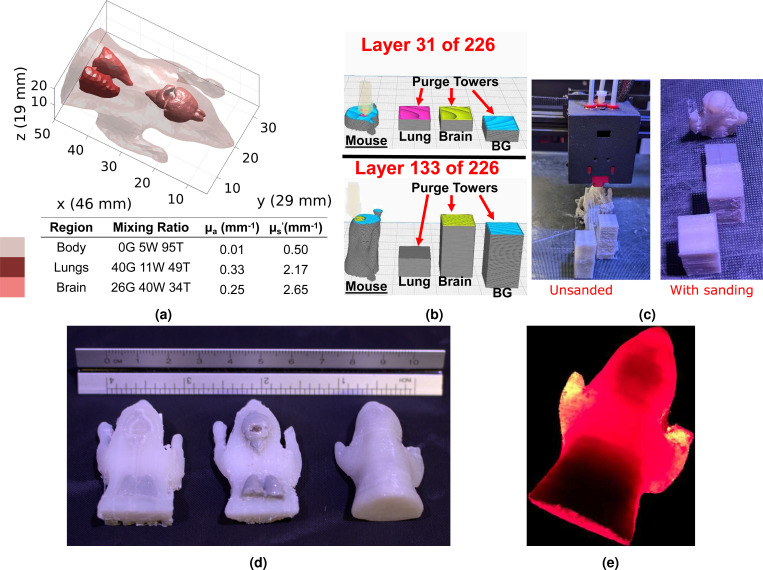
Fabrication of a complex shaped 3-D optical phantom derived from the Digimouse atlas. In (a), the schematic of the digital model, together with the optical properties for each labeled region, is reported. In (b), two sample 3-D slicer renderings of the printing process, one aligned with the lung region, and the other aligned with the brain region, are shown to demonstrate the purge tower configurations. An animation showing the extruder travel paths can be found in Visualization 1. Furthermore, photos of the phantom during and after the printing are shown in (c-d). In (d), two partially phantoms are placed alongside with the completed phantom, showing the complex inclusion shapes. Lastly, in (e), the trans-illumination image of the phantom is shown.

In Fig. 5, we show the phantom schematic in 5a and photos in 5c of the printed Digimouse phantom before and after sanding. In Fig. 5d, we show three photos of different variants of the Digimouse phantom to expose the complex inclusion boundaries. To further illustrate the fabrication process of this complex phantom, we also report slicer 3-D rendering of the phantom and the purge towers in Fig. 5b at a sample slice #31 when printing the lung region, and another slice at #133 when printing the brain region. An animation generated by the slicer showing the travel paths of the extruder can be found in Visualization 1. Finally, in Fig. 5e, we show a transilluminated image of the completed Digimouse phantom to qualitatively demonstrate the high absorption anticipated at the brain and lung regions.

### 3.5 Prototype DOI QA phantom

**Figure 6.**
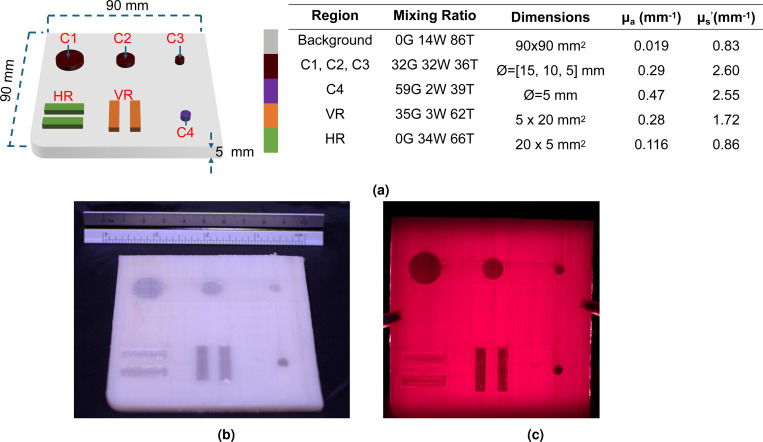
Diffuse optical imaging (DOI) quality assurance (QA) phantom schematic and results. In (a), we show a design schematic of our DOI QA phantom as well as the key inclusion regions. The targeted optical property for each inclusion along with their dimensions are also included. In (b), we show a picture of the inclusion-exposing side of the printed phantom. In (c), we show a trans-illumination image of the printed phantom.

A schematic and a color photo of the inclusion-exposed side of the 3-D printed DOI QA phantom prototype (Section 2.8) are shown in Figs. 6a and 6b, respectively. This print takes about 7 hours using the ZoneStar printer, consuming about and 59 g of filament materials. All printed inclusions are visually discernible at the designed location. A trans-illumination image of this phantom is shown in Fig. 6c, further attesting that the printed inclusion contrasts visually correlate with the designed contrasts as shown in Section 2.8. We want to point out that the trans-illumination image also demonstrates some noticeable printing artifacts, such as black lines between inclusions as a result of filament stringing and the slightly darkened vertical and horizontal shades extended from the inclusion due to smearing of the extruder.

### 3.6 Complex heterogeneous DOT phantom

**Figure 7.**
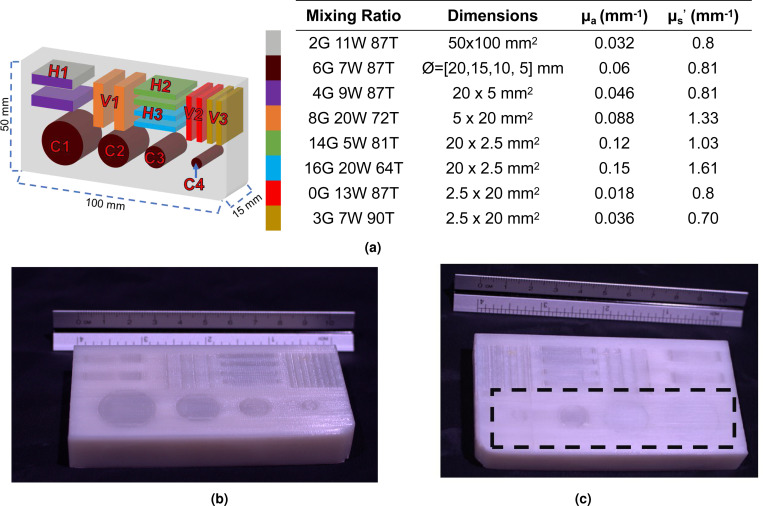
Fabricating complex diffuse optical tomography (DOT) phantom. In (a), we show a schematic of the phantom design as well as the eight different mixing combinations corresponding to each pair of optical properties present over the entire model. The two largest cylindrical inclusions (C1 and C2), extend 13 mm and 14 mm respectively, while all other inclusions extend the full 15 mm across the thickness of the phantom. In (b), we show one side of the printed phantom where all inclusions directly visible. In (c), we show the reversed side of the printed phantom. A black dashed rectangle is placed around the cylindrical inclusions (C1-C4) with the C1-C2 appearing faded as they are embedded underneath the surface.

A photo of the 3-D printed DOT phantom is shown in Fig. 7b. The total printing time for this phantom is around 30 hours, using a total of 221 grams of gray, white and translucent filament materials. Similar to the QA phantom above, all inclusions show visually discernible boundaries along the side where they are exposed (Fig. 7b). In Fig. 7c, the photo taken at the opposite side of the phantom show similar inclusion boundaries, except the two largest cylindrical inclusions, C1 and C2. This is expected because those 2 inclusions are not directly exposed on the reversed side. In this print, we also observe similar streaking and smearing artifacts as showed in the printed QA phantom above; the infills of the two small cylindrical inclusion (C3/C4) also appear to be uneven compared to other inclusions. This is likely because of the lower purging volume.

## 4 Discussion

From the results shown in Figs. 2e and 2g, the SFDI-measured $\mu_{a}$ and $\mu_{s}^{\prime }$ values across the 16× titration phantoms follow the expected bi-linear relationships with gray $\left(R_{g}\right)$ and white filament $\left(R_{w}\right)$ mixing ratios. This strong linear relationship is also further validated by the reported $R^{2}$ values obtained from the linear fitting of both optical properties. The fitted linear coefficients suggest that the absorption coefficient $\mu_{a}$ is largely modulated by the mixing ratio of the gray-colored filament $\left(R_{g}\right)$, as expected. In comparison, the reduced scattering coefficient, $\mu_{s}^{\prime }$ is modulated by both the gray and white filaments, with a roughly equal weight. The translucent filament also appears to set a lower bound of 0.0056 mm^−1^ for $\mu_{a}$ and 0.35 mm^−1^ for $\mu_{s}^{\prime }$. The theoretical range of optical properties based on our linear-mixing model and selected gray/white/translucent filaments are from 0.0056 mm^−1^ $\left(R_{g}=R_{w}=0\right)$ to 0.7856 mm^−1^ ($R_{g}=100$, $R_{w}=0$) for $\mu_{a}$ and from 0.35 mm^−1^ $\left(R_{g}=R_{w}=0\right)$ to 3.95 mm^−1^ ($R_{g}=100$) for $\mu_{s}^{\prime }$. These values cover a broad range of absorption and scattering coefficients present in diverse biological tissues with only a few exceptions. One such exceptions is the low scattering tissue such as cerebrospinal fluid (CSF) in the brain or synovial fluid in the joints^64^. When printing CSF or synovial fluid is necessary, one could consider using alternative filament colors such as a clearer PLA or use a slightly lower infill ratio for printing to create less absorptive phantoms. This will likely lower both the absorption and scattering coefficient lower bounds. Our printing technique allows for the generation of complex shaped voids to simulate air cavities similar to those seen in the lungs and nasal cavities. However, when printing voids with complex shapes, the remaining unused filament channel can be used to print water-soluble supporting materials that can allow for proper printing of the desired structure while still allowing for artifact-free smooth surfaces by dissolving the support structures.

From the results shown in Fig. 3d, the average error of the 5× validation prints relative to the fitted linear-mixing models is less than 15% for both $\mu_{a}$ and $\mu_{s}^{\prime }$. This suggests that our hypothesized linear-mixing models for $\mu_{a}$ and $\mu_{s}^{\prime }$ appear to hold across a wide range of $R_{g}$ and $R_{w}$ settings. This result also demonstrates a generalizable approach to extend our filament-mixing optical property characterization from the specific filament types used in this study to other off-the-shelf filaments available to the users. When a new set of filaments are used, one can repeat our above experiments and obtain the corresponding linear-mixing models, based on which new phantoms can be designed.

The filament-mixing characterization prints shown in Figs. 4d-4f, as well as the QA phantom shown in Fig. 6 demonstrate the ability of creating spatially tunable absorption and scattering heterogeneities using our proposed 3-D printing based phantom fabrication method. All of these prints yield clear color transitions in single-axial or 2-D heterogeneous domain layouts. The color images show clear visual differences due to the modulated absorption/scattering across different printed segments. Within each square shaped tile in Benchmark #3, the surface color appears to be relatively uniform, suggesting the stability of the extruder for producing uniform optical properties at each programmed $R_{g}/R_{w}/R_{t}$ setting. Furthermore, the prints generated from Benchmarks #2 and #3 also demonstrate the ability of the printer to create potentially arbitrarily complex topological structures. We want to particularly highlight that printing small heterogeneous sample with programmable patterns and optical properties can be relatively fast to print. For example, the 9-material characterization palette print shown in Fig. 4f took 4.5 hours to complete using our ZoneStar printer while the QA phantom in Fig. 6 took roughly 7 hours to complete. These simple benchmark prints can be quite useful for testing and characterizing reflection-based DOI imaging systems, including SFDI instrumentation. The DOI QA phantom shown in Fig. 6 represents, to the best of our knowledge, the first attempt of designing a widely accessible and reproducible optical imaging phantom that can be independently fabricated across labs.

For the purpose of a simplified visual assessment, in the QA phantom design shown in Fig. 6, we set the inclusion optical properties to a high absorption value, mimicking highly absorbing tissues such as blood vessels. We want to highlight that such phantoms can also be used to simulate tissues of reduced absorption and scattering contrasts if one can leverage the linear scalability of $\mu_{a}$ and $\mu_{s}^{\prime }$. This is because both $\mu_{a}$ and $\mu_{s}^{\prime }$ are length-dependent. In the diffusion regime, shrinking the source/detector separation by a factor of 2 is equivalent to reducing both $\mu_{a}$ and $\mu_{s}^{\prime }$ by a factor of 2. Similarly, doubling the source/detector separation over a domain with doubled $\mu_{a}$ and $\mu_{s}^{\prime }$ is expected to produce equivalent measurements to the unscaled domain. This optical property scaling approach offers additional flexibility. It can be particularly useful for compensating for the limited color-range of the selected filaments, or the limited color-mixing precision of the extruder. In such cases, linearly-scaled optical properties can be used as long as one also scales the corresponding source/detector separation.

The Digimouse phantom shown in Fig. 5 further showcases the versatility of our 3-D printing based technique for creating heterogeneous phantoms with complex 3-D boundaries. Although both inclusions, the brain and the lung, are not directly exposed from the completed print, we can clearly see from the photos of the partially printed phantom and inclusion-extruded prints in Fig. 5d that the printed inclusions capture the complex 3-D shapes of both regions, printed with the more absorbing materials as expected. In this particular print, we align the Digimouse’s torso in the z-axis of the printer. The primary consideration for choosing this printing direction is to limit the maximum number of unique mixing ratios in all z-slices. This helps minimize color transition along each slice and the associated stringing and smearing artifacts. For z-slices along the span of the brain region, only two color transitions are needed. All slices below the top-most position of the lung region are printed with 3 purge towers, even though the brain material is not used in the phantom region.

The DOT phantom shown in Fig. 7 is quite challenging to print and provides a valuable “stress-test” to the printer and the proposed technology. While the ZoneStar printer we use has a maximum printing volume of 30 × 30 × 40 cm^3^, it is mostly designed to create prints with sparse infills. In comparison, this 10 × 1.5 × 5 cm^3^ DOT phantom has 110% infill and takes over 23 hours to complete. Several attempts had been made for completing this print. Some of the failed attempts were caused by tangled filaments that failed to feed the extruder; other failed prints were caused by inconsistent extruder feeding and skipping. Overall, we found that low-cost multi-filament printers, such as the one we use in this work, require that the operator monitors the system from time to time to mitigate issues throughout the printing process. Regardless, the completed phantom does exhibit sophisticated spatial heterogeneous structures and tunable optical proprieties that are substantially more advanced compared to other previously reported phantom fabrication methods. The inclusion arrangements in this phantom also make it well suited for characterizing reflection-based DOT or widefield spectroscopy systems, including SFDI imaging or tomography systems. In addition, this phantom can also be used to characterize transmission-based widefield DOT systems^65^ or single-pixel camera (SPC) systems^66,67^.

Despite the significant expansion of the complexity and versatility of optical phantom fabrication enabled by our proposed method, the prints shown in Section 3 also suggest a number of limitations that deserve to be further investigated. First, from the trans-illumination image shown in Fig. 6c and color images shown in Figs. 7b and 7c, we can see that some portion of the background region contains small traces of higher absorption mixed filaments from the inclusion. We believe that these small defects are results of “stringing” effect from the extruder. The stringing effect in 3-D printing typically refers to the thin threads of filaments that are stretched by the undesired residual filament materials at the nozzle tip along the movement paths. The stringing effect is related to many factors, such as extruder temperature settings, travel speed, extrusion motor retraction settings, and filament quality. It is a common issue appearing in many FDM 3-D printers. There are well documented heuristics for minimizing the stringing effect^68^, but many of these heuristics are printer and filament-specific, often requiring manual tuning^68^. It is plausible that other color-mixing extruders as well as other filament types are less prone to stringing issues and thus could mitigate such defects. Reorienting the phantom to minimize switching between filament mixing ratios could also be highly effective in reducing such defects.

Secondly, the target DOT optical properties we tested in this work are largely focused on highly scattering diffusive media, similar to those found in biological tissues. In such cases, the choice of infill patterns, i.e. the routing orientations of the extruder when filling the solid homogeneous regions in each printed layer, does not appear to significantly alter light diffusion propagation. However, when printing low-scattering or transparent tissue regions, it is perceivable that the shape of the extruded filament in each layer may cause multiple reflection events between extruded filament boundaries, known as the “light-piping” effect. In this work, we did not characterize this behavior in a low-scattering medium, but we are interested in further investigating this effect in our future experiments and through simulation studies. Similarly, the terraced surface shape/texture of the print due to the layer-by-layer printing process may also have an impact on light incidence and back-scattering. Characterizing this terraced boundary effect would also inform important future studies.

Thirdly, despite the commercial availability of a few inexpensive multi-filament mixing printers, the reliability of these printers, especially for making solid prints with high (>100%) infill ratios, can cause manufacturing difficulties. Based on our experience of using our ZoneStar M6V5 4-in-1 extruder, we found that the low-cost ($50 USD) extruder head occasionally suffers from clogging and extruder gear skipping (i.e. failing to uniformly feed filaments). Fortunately, due to the relatively low cost, replacing the defective extruder head would often solve such issues. Proper maintenance and fine-tuning of the printer frame and the extruder head, including manual optimization of printing speed, extrusion gear maintenance, retraction settings and extruder/bed temperature settings should be applied to any printer to allow for increased system reliability.

Although we acknowledge some of the limitations and quality defects observed in our limited printed phantom samples, we also must reiterate that filament-mixing based 3-D printers are only recently beginning to gain increased market prominence and that we only tested our approach at an inexpensive model (the Z9V5 Pro printer costs only about $500) to show initial proof-of-concept while allowing for widespread adoption among the research community. However, recent technologies such as multi-jet printers utilizing photo-curing in conjunction with dyes use similar mixing methods and could theoretically be leveraged for DOI phantom fabrication albeit at significantly higher costs (>$100,000) while offering potential significant improvements to phantom quality. The usage of these sophisticated printing technologies thus remain an area of interest for further studies especially as these technologies become more affordable and commercially available.

We also acknowledge that the optical properties of commercially available PLA filaments are expected to be vendor- or batch-dependent, and our fitted linear-mixing model may only be applicable for the specific brand and batch of filaments that we have used. However, we also want to highlight that the titration methods we used to characterize the linear-mixing model can be readily applicable to any filament of other vendors as well as any other colors. In addition, the same process of characterizing optical properties via a titration study can be extended to accommodate other commonly used filament materials such as nylon, Thermoplastic polyurethane (TPU), ABS, and Polyethylene Terephthalate Glycol (PETG) to allow for enhanced mechanical flexibility as well as chemical and thermal stability if a specific research application is desired. The titration “rulers” are relatively small and fast to print. Repeating such a characterization procedure is highly recommended before fabricating complex phantoms. For more stable and target-tissue specific optical properties, it is also possible to use commercially available filament extruder machines^40^ to create more chemically stable filaments using standardized compounds. Although this would require an additional manual step in phantom fabrication, only three parent filaments would need to be created. The subsequent mixing process would automatically combine these filaments, offering significant benefits for manufacturing complex phantoms.

## 5 Conclusion

In summary, we have reported a highly versatile method to design and directly 3-D print complex heterogeneous DOI optical phantoms with arbitrary complexity and 3-D shapes. Our method utilizes commercially available gray, white and translucent PLA filaments as well as off-the-shelf color mixing based FDM 3-D printers, thus providing a broadly accessible and inexpensive approach for fabricating complex DOI phantom across different labs and studies. We have hypothesized a linear-mixing model for quantitatively tuning the absorption and reduced scattering coefficients with respect to the volume ratios of the filaments being mixed, and experimentally validated this model, showing excellent linearity across large ranges of target $\mu_{a}$ and $\mu_{s}^{\prime }$ values relevant to biological tissue imaging. We have also showcased a number of complex phantom designs, including a Digimouse phantom, a DOI QA phantom and a complex DOT phantom prototype, to further demonstrate the versatility and easy-programmability of our 3-D printing approach. While the printer and the sample prints we reported in the Results section still present some minor defects and robustness issues, we believe that these issues can be further mitigated with the continual development of filament-mixing printers and extruders. Further refinement of the printing strategies, such as extruder routing directions, purging tower designs, or even adding additional purging approaches such as purge chutes, could potentially enhance the quality of the print. In addition, our reported 3-D printing based phantom fabrication platform can be easily extended towards characterizing other imaging contrasts, such as phosphorescence/fluorescence, among others^52^. The ability to design, and in-house fabricate complex heterogeneous phantoms with full programmability of inclusion locations, contrasts and shapes would greatly facilitate the characterization, cross-validation, comparison, and standardization of NIR based imaging or spectroscopy systems, promoting reproducible research and reusable study data sharing among the DOI research community.

## Figures and Tables

**Table 1.** Target filament mixing ratios used for (a) 16× titration prints and (b) 5× validation prints.

**(a)**			

Sample ID	Filament Mixing Composition		
	Gray %	White %	Translucent %
**1-A**	5	10	85
**1-B**	9		81
**1-C**	13		77
**1-D**	17		73
**2-A**	5	15	80
**2-B**	9		77
**2-C**	13		73
**2-D**	17		69
**3-A**	5	20	75
**3-B**	9		71
**3-C**	13		67
**3-D**	17		63
**4-A**	0	5	95
**4-B**		10	90
**4-C**		15	85
**4-D**		20	80

**(b)**			

Sample ID	Filament Mixing Composition		
	Gray %	White %	Translucent %
**V-1**	15	19	66
**V-2**	15	12	73
**V-3**	11	13	76
**V-4**	7	15	78
**V-5**	3	15	82

## Data Availability

The finalized Cura printer profile as well STL meshes of all heterogeneous phantoms can be found at https://github.com/COTILab/Printable_DOI_Phantoms.
